# Nutrition Interventions Integrated into an Existing Maternal, Neonatal, and Child Health Program Reduce Food Insecurity Among Recently Delivered and Pregnant Women in Bangladesh

**DOI:** 10.1093/jn/nxy249

**Published:** 2019-01-11

**Authors:** Edward A Frongillo, Phuong H Nguyen, Tina Sanghvi, Zeba Mahmud, Bachera Aktar, Silvia Alayon, Purnima Menon

**Affiliations:** 1 Department of Health Promotion, Education, and Behavior, University of South Carolina, Columbia, SC; 2 Poverty, Health and Nutrition Division, International Food Policy Research Institute, Washington, DC; 3 FHI 360, Washington, DC; 4 BRAC, James P Grant School of Public Health, BRAC University, Bangladesh; 5 Save the Children, Washington, DC

**Keywords:** food insecurity, community mobilization, interpersonal communication, pregnancy, antenatal care

## Abstract

**Background:**

Antenatal care may be a means to reduce food insecurity in pregnancy and postpartum periods.

**Objective:**

With the use of a cluster-randomized design, we tested whether participation in nutrition-focused antenatal care intending to improve household knowledge about the importance of nutrition for pregnant and lactating women and encourage allocation of household resources to ensure sufficient quality and quantity of foods, without providing food assistance, would reduce household food insecurity.

**Methods:**

Alive & Thrive integrated nutrition interventions into an existing Maternal, Neonatal, and Child Health (MNCH) program in Bangladesh. The nutrition-focused MNCH package was delivered in 10 subdistricts through antenatal care visits with the use of interpersonal communication, community mobilization, and monitoring of weight gain, aiming to improve maternal diet quality, quantity, and micronutrient intake during pregnancy and breastfeeding. The package included components that could reduce food insecurity, measured using the Household Food Insecurity Access Scale. To examine the impact of the nutrition-focused MNCH package compared with the standard MNCH package, we used linear and multinomial logit regression models, adjusted for subdistrict clustering, to test differences at endline in items, domains, and categories of food insecurity, after first confirming no differences at baseline.

**Results:**

At baseline, nearly half of households were food insecure. At endline, the groups differed in food insecurity, whether expressed as items, domains, or categories, with food insecurity in the nutrition-focused MNCH group 22 percentage points lower than in the standard MNCH group and 20 percentage points lower than at baseline.

**Conclusions:**

Participation in nutrition-focused antenatal care reduced household food insecurity among recently delivered and pregnant women. Integration of social and behavioral nutrition interventions into antenatal care with components that promote food security provides a potentially effective means to reduce food insecurity, without incurring high costs of providing supplemental food, in populations where limited resources can be directed towards accessing adequate and appropriate foods. Registered at clinicaltrials.gov as NCT02745249.

## Introduction

Food insecurity has pervasive consequences across the life course (1). Food insecurity is a particular concern for pregnant women and lactating mothers. Women who are pregnant have higher nutritional needs than when not pregnant to meet the high demands of the growing fetus. Furthermore, because pregnancy is physically demanding, pregnant women may have greater difficulty acquiring and preparing nutritious food and have lower capacity to work, exacerbating food insecurity and its consequences (2). Food insecurity may be associated with risk of low birth weight and some birth defects (3, 4). Food insecurity among pregnant women and mothers is associated with stress, anxiety, and depressive symptoms, which may in turn affect parenting practices and infant development (2, 4–8).

Antenatal care is a common healthcare intervention for pregnant women across the globe, although coverage is inadequate in many countries, especially in sub-Saharan Africa and South Asia where fewer than half of pregnant women receive ≥4 of the recommended 8 visits with skilled health personnel (9). Antenatal care provides 4 basic functions for pregnant women: confirming health of the fetus and woman; preventing and monitoring medical complications; building supportive provider relationships; and educating and preparing for the pregnancy, delivery, and postpartum periods (10). Antenatal care also provides a potentially important means through which to achieve complementary goals for well-being and health through use of risk assessment, health promotion, and clinical and psychosocial interventions (11). For example, as shown in a prior quasi-experimental design, antenatal care may be a means through which to reduce food insecurity in the pregnancy and postpartum periods through behavioral and social interventions that do not provide food or money (12).

Other evidence that food security can be improved through behavioral and social intervention comes from a study in Honduras and studies of the Expanded Food and Nutrition Education program for low-income populations in the United States. In Honduras, a nutritional-counseling intervention delivered by peers to antiretroviral therapy patients with diverse nutritional status was evaluated through use of a pre- and posttest design. The intervention was associated with increased dietary quality and decreased food insecurity (13). The program in the United States was found to be cost-beneficial, with the benefits including reduced food expenditures, higher intakes of nutrients, adding less salt, reading nutrition labels more often, and running out of food at the end of the month less often (14). Food insecurity decreased significantly more in graduates than in terminated participants, with a dose-response relation between the number of lessons received and decreases in food insecurity (15). In a randomized design, a behavioral score constructed from items on diet quality, food safety, food security, and food resource management showed differential improvement over 2 mo (16). Also, a cross-sectional survey showed that greater financial management skills in households was associated with less food insecurity, suggesting that improving these skills could reduce food insecurity (17).

The Alive & Thrive initiative worked with BRAC, a large nongovernmental organization, to integrate multiple nutrition interventions into BRAC's existing Maternal, Neonatal, and Child Health (MNCH) program in Bangladesh, a country in which maternal and child undernutrition is pervasive (18). This nutrition-focused MNCH package improved maternal dietary diversity and micronutrient supplement consumption during pregnancy, improved exclusive breastfeeding practices, and increased the frequency of monitoring of maternal weight gain compared to the standard MNCH package (18). The nutrition-focused MNCH package intended to improve knowledge of decision-makers in the household about the importance of nutrition for pregnant and lactating women and to encourage allocation of resources to ensure sufficient quality and quantity of foods in the household. Interpersonal communication and community mobilization involving men and other community members focused on improving women's diets during pregnancy and the early postnatal period through greater purchase and availability of foods in the household. We hypothesized that these actions to increase the purchase and availability of foods in the household would lead, in turn, to greater household food security as reported by women.

## Methods

### 

#### Intervention

The nutrition-focused MNCH package was started in 10 subdistricts in August 2015 and continued until mid-2017. The intervention package, which included several components that could reduce food insecurity, was delivered through antenatal care visits with the use of interpersonal communication, community mobilization, and monitoring of weight gain, aiming to improve maternal nutrition by increasing maternal diet quality, micronutrient intake, and breastfeeding practices (18).

For interpersonal communication, frontline workers (called Shasthya Kormi) and health volunteers (called Shasthya Sebika) were trained to counsel pregnant and recently delivered women (with children aged <6 mo) during regularly scheduled monthly antenatal care visits at the household. The frontline workers demonstrated a specific diet plan (both quality and quantity), provided free iron and folic acid and calcium supplements, measured weight, counseled on resting, engaged other family members to support pregnant women, and encouraged husbands and family members to make food readily available in the household, and, in turn, encouraged pregnant women to follow the recommended diet. The diet-planning sessions discussed consumption of nutrient-dense foods during pregnancy, such as fish or meat, egg, milk or milk products, green leafy vegetables, and yellow or orange fruits and vegetables in addition to rice and thick lentils. Appropriate portion sizes for these foods were discussed through use of counts, pictures, and a 250-mL bowl.

Community mobilization involved husbands’ forums and interactive video shows in the community. Husbands of pregnant women were invited to attend 2 forums, 1 each in the second and third trimesters of pregnancy. The husbands’ forums provided information about the importance of proper nutrition for women during pregnancy for the development of the fetus and importance of nutrition in the postpartum period; encouraged husbands to purchase diversified nutritious foods for their wives; and involved the husbands in ensuring intake of the recommended quantity of diversified foods, iron and folic acid tablets, and calcium tablets by wives. Video shows and interactive communication were carried out in the community on multiple nutrition topics, targeting pregnant women, their husbands and family members, local elites, village doctors, and government health workers. A theme in video shows featuring husbands and mothers-in-law was the importance and priority of ensuring the availability and intake of diverse and nutritious foods by pregnant women, even if necessitating trade-offs with meeting other needs or wants. For example, 1 video showed a woman giving her son some saved money to buy fish for his pregnant wife, and another video showed that a husband should cut down other costs for some time to buy nutritious food for his pregnant wife and that buying nutritious foods for pregnant women is the best use of saved money.

#### Study design and participants

A cluster-randomized, nonblinded design was used to evaluate the impact of the nutrition-focused MNCH compared to the standard MNCH packages in Bangladesh (18). Standard MNCH was provided in the control areas where home visits were less frequent, included much less nutrition content or emphasis, provided micronutrient supplements upon payment, and had no community mobilization.

**FIGURE 1:**
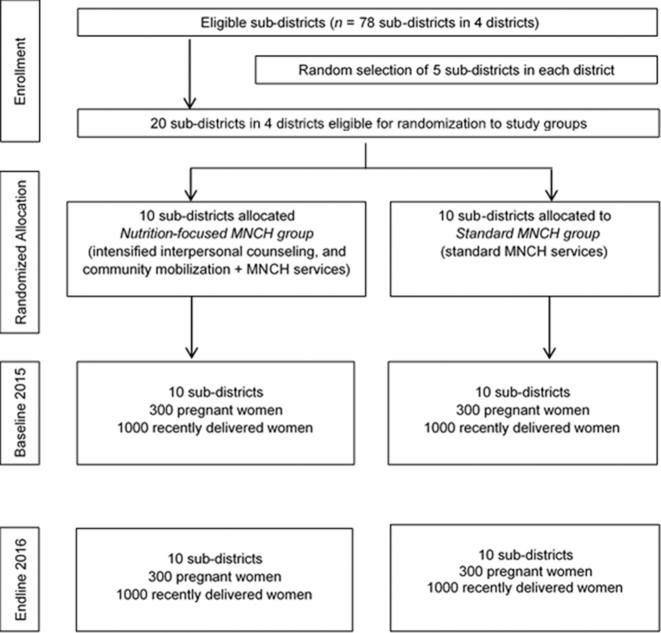
Trial profile. MNCH, Maternal, Neonatal, and Child Health.

Twenty subdistricts were randomly assigned to either the nutrition-focused MNCH or the standard MNCH package (**Figure 1**). Cross-sectional household surveys were conducted at baseline (2015) and endline (2016) in the same communities and at the same time of year (June to August). A total of 2000 recently delivered women with children aged <6 mo (1000/intervention group) and 600 pregnant women in the second and third trimesters of pregnancy (300/intervention group) were surveyed at each survey round. To obtain the samples, within each subdistrict, 5 unions and 2 villages within each union were randomly selected to yield a total of 200 villages. Villages were a mean size of 250 households. Within each village, a household census was conducted at baseline and endline to list pregnant women and mothers with infants <6 mo of age. We selected households for surveys with the use of systematic sampling beginning with a random seed as a starting point to yield the desired sample size per cluster.

#### Measurements

Household food insecurity was measured by asking recently delivered and pregnant women to respond to the questions from the Household Food Insecurity Access Scale (19), which had 9 items related to the household's experience of food insecurity in the past 30 d. These items aimed to capture 3 main domains of household food insecurity: anxiety and uncertainty about the household food supply (1 item), insufficient quality (3 items), and insufficient quantity and its physical consequences (5 items). We calculated at baseline and endline the percentage of households that *1*) responded “yes” to a specific occurrence question, *2*) responded “yes” to any of the conditions in a specific domain, and *3*) were categorized as food-secure and mild, moderate, and severe food-insecure following the steps described in the published guide (19).

Women were asked at both baseline and endline whether anyone in the household received cash, food, or other types of social assistance, and also whether anyone in the household received a microcredit loan. Husbands were asked at endline if they were able to regularly (i.e., ≥3 times/wk) buy each of 12 foods presented on a list.

We measured several maternal and household characteristics. Maternal variables measured were mother's age, religion, education, and occupation. Household variables measured were household size, number of children, and socioeconomic status, which was created by principal components analysis with the use of a set of items related to ownership of property (20).

#### Ethical approval

The institutional review boards at the International Food Policy Research Institute and the Bangladesh Medical Research Council both approved this study. All women were provided with detailed information about the study in writing and verbally at recruitment, and written informed consent was obtained.

#### Statistical analysis

Analyses were stratified by recently delivered and pregnant women. Baseline differences between the 2 intervention groups in maternal and household characteristics were examined using linear regression models for continuous variables or logit regression models for categorical variables, accounting for subdistricts as a random effect through use of a cluster sandwich estimator (21).

To examine the impact of the nutrition-focused MNCH package in comparison with the standard MNCH package, we used linear (for items and domains) and multinomial logit (for categories) regression models in an intent-to-treat analysis to test differences at endline after first confirming that there were no differences at baseline. Linear regression was used for items and domains because the coefficients estimated differences in prevalence; linear and logit models provide similar results when the distribution of the outcome is not extreme (22, 23). We accounted for variation among subdistricts through use of a cluster sandwich estimator, testing the coefficients with the standard error and denominator degrees of freedom that reflected the subdistrict level. Data analyses were intent-to-treat, and were performed with the use of Stata 14. *P* values <0.05 were considered to be statistically significant.

For a robustness check, we re-ran analyses accounting for baseline measures of food security. The results were essentially the same; accounting for baseline measures slightly attenuated some differences between nutrition-focused and standard MNCH groups, but also decreased precision of estimates. Also, to examine possible bias in responses from social desirability, a subset of 5 questions that we adapted and used previously for this purpose was administered to women at endline (24, 25). The items addressed whether a woman gives up doing something because she does not think she has the ability, feels like not listening to people even if she knows they are right, gets irritated or annoyed by people who ask her to do something for them, is courteous to people who are unpleasant, and is willing to admit a mistake when she makes it. A social desirability score was created by adding up the number of responses indicative of a propensity to provide socially desirable answers, with a score of 0–2 being considered low, 3 as medium, 4 as high, and 5 as very high social desirability. The food insecurity categories were tabulated by these score values to examine differences in reported food insecurity.

## Results

The nutrition-focused and standard MNCH groups were similar in terms of maternal and household characteristics at baseline for both recently delivered and pregnant women (**Table 1**). The mean age of women was 24 y, and most were not working outside the home. For schooling, the women had completed a mean of 6 y, but >10% of women had no schooling, and >80% did not complete high school. Households had a mean of 4–5 members.

**TABLE 1:** Characteristics of the sample at baseline by intervention package for recently delivered and pregnant women^1^

	Recently delivered women		Pregnant women	
	Nutrition-focused MNCH (*n* = 1000)	Standard MNCH (*n* = 1000)	Nutrition-focused MNCH (*n* = 300)	Standard MNCH (*n* = 300)
Maternal characteristics				
Age of respondent mother, y	24.7 ± 5.4	24.2 ± 5.6	24.3 ± 5.6	23.7 ± 5.6
Duration of pregnancy, mo	—	—	6.2 ± 1.5	6.2 ± 1.5
Schooling, years completed	6.0 ± 3.4	6.0 ± 3.5	6.0 ± 3.2	5.9 ± 3.3
Education level				
Never attended school	10.4	12.8	11.7	12.7
Primary school (grades 1–5)	36.4	33.9	30.0	33.0
Middle school (grades 6–9)	37.9	37.9	46.7	42.7
High school or higher	15.3	15.4	11.7	11.7
Occupation				
Household work/housewife	89.4	90.3	89.3	87.3
Self-employment	8.1	6.6	8.3	8.3
Other	2.5	3.1	2.3	4.3
Religion				
Muslims	93.6	93.5	93.3	93.3
Hindus	6.4	6.5	6.7	6.7
Household characteristics				
Household size, *n*	5.2 ± 1.9	5.0 ± 1.8	4.0 ± 1.7	4.1 ± 1.7
Number of children <5 y of age, *n*	1.3 ± 0.5	1.3 ± 0.5	0.3 ± 0.5	0.3 ± 0.5
Socioeconomic index^2^	−0.06 ± 0.99	−0.06 ± 0.96	−0.03 ± 0.84	0.15 ± 0.98

1 Values are means ± SDs or percentages. MNCH, Maternal, Neonatal, and Child Health.

2 Socioeconomic index was constructed with the use of principal components analysis with variables on ownerships and assets, and was standardized with mean 0 and standard deviation 1.

**FIGURE 2:**
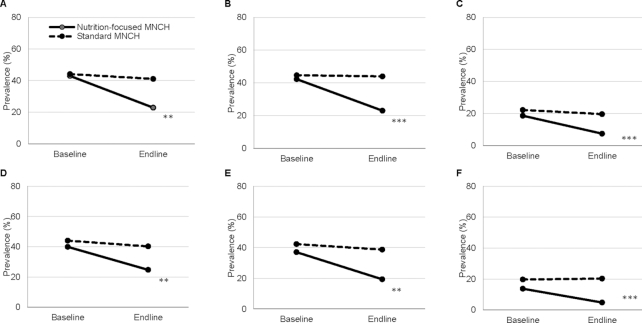
Household food insecurity among RDW and PW, by survey round (i.e., baseline in 2015 and endline in 2016) and intervention package (i.e., nutrition-focused and standard MNCH), for domains: (A) RDW-anxiety and uncertainty about the household food supply, (B) RDW-insufficient quality, (C) RDW-insufficient food intake, (D) PW-anxiety and uncertainty about the household food supply, (E) PW-insufficient quality, and (F) PW-insufficient food intake. **P < 0.01, ***P < 0.001. MNCH, Maternal, Neonatal, and Child Health; PW, pregnant women; RDW, recently delivered women.

**FIGURE 3:**
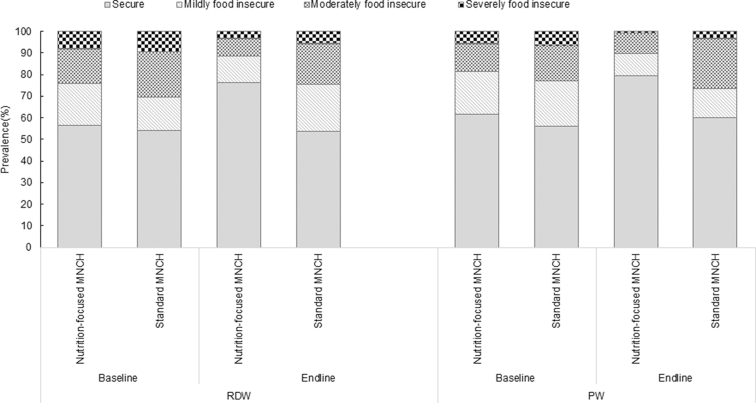
Categories of household food insecurity among RDW and PW, by survey round (i.e., baseline in 2015 and endline in 2016) and intervention package (i.e., nutrition-focused and standard MNCH). A food-secure household experienced none of the food insecurity (access) conditions, or just experienced worry, but rarely. A mildly food-insecure household worried about not having enough food sometimes or often, and/or was unable to eat preferred foods, and/or ate a more monotonous diet than desired. A moderately food-insecure household sacrificed quality more frequently, by eating a monotonous diet or undesirable foods sometimes or often, and/or started to cut back on quantity by reducing the size of meals or number of meals. A severely food-insecure household graduated to cutting back on meal size or number of meals often, and/or experienced any of the 3 most severe conditions (running out of food, going to bed hungry, or going a whole day and night without eating). MNCH, Maternal, Neonatal, and Child Health; PW, pregnant women; RDW, recently delivered women.

The 2 groups were similar at baseline for food insecurity whether expressed as separate items, domains, or categories (**Table 2** and **Figures 2** and **3**). At baseline, nearly half of households were categorized as food-insecure (i.e., mild, moderate, or severe food-insecure).

**TABLE 2:** Items of household food insecurity among recently delivered women and pregnant women, by survey round and intervention package^1^

	Baseline		Endline	
	Nutrition-focused MNCH	Standard MNCH	Nutrition-focused MNCH	Standard MNCH
Recently delivered women^2^				
Worry that your household would not have enough food	43.0	44.1	22.8	41.1**
Not able to eat the kinds of foods you preferred because of a lack of resources	40.4	42.7	20.9	40.7**
Eat just a few kinds of food day after day because of a lack of resources	34.5	36.6	16.4	34.5***
Eat food that you did not want to eat because of a lack of resources to obtain other types of food	28.6	30.2	12.7	32.7***
Eat a smaller meal than you felt you needed because there was not enough food	17.9	21.1	6.9	19.0***
Eat fewer meals in a day because there was not enough food	6.4	8.1	2.7	4.6
No food at all in your household because there were no resources to get more	7.3	8.4	2.6	5.1**
Go to sleep at night hungry because there was not enough food	4.7	3.5	1.6	2.5
Go a whole day without eating anything because there was not enough food	0.2	0.6	0.3	0.2
Pregnant women				
Worry that your household would not have enough food	40.0	44.0	24.7	40.3**
Not able to eat the kinds of foods you preferred because of a lack of resources	35.0	40.7	18.3	35.7**
Eat just a few kinds of food day after day because of a lack of resources	31.7	33.3	16.3	31.7**
Eat food that you did not want to eat because of a lack of resources to obtain other types of food	25.0	29.3	11.0	30.3**
Eat a smaller meal than you felt you needed because there was not enough food	12.3	17.0	4.0	19.3***
Eat fewer meals in a day because there was not enough food	3.3	3.0	0.7	3.7*
No food at all in your household because there were no resources to get more	5.3	6.3	0.7	3.3*
Go to sleep at night hungry because there was not enough food	2.0	2.0	0.0	2.3*
Go a whole day without eating anything because there was not enough food	0.3	1.0	0.3	0.7

1 Recently delivered women, *n* = 1000/intervention; pregnant women, *n* = 300/intervention. Values are percentages. Testing was done for difference between nutrition-focused MNCH and standard MNCH at endline: *P < 0.05, **P < 0.01, ***P < 0.001. MNCH, Maternal, Neonatal, and Child Health.

2 Average interval since delivery was 93 d.

At endline, with the exception of the most severe item measured on going a whole day without food (which was rarely affirmed), the 2 groups were different for food insecurity for each separate item and each domain, as well as among the categories, with the prevalence of food insecurity in the nutrition-focused MNCH group lower than that for the standard MNCH group and lower than that at baseline for either group (Table 2 and Figures 2 and 3). For example, among recently delivered women at endline, the prevalence of anxiety and uncertainty about the household food supply, insufficient quality, and insufficient intake in the nutrition-focused MNCH group were 22.8%, 23.0%, and 7.5%, respectively, much lower than those in the standard MNCH group (41%, 44%, and 20%, respectively). Overall, the prevalence of any food insecurity was 22.3 and 19.7 percentage points lower for the nutrition-focused MNCH group compared to the standard MNCH group at endline (*P* < 0.01) for recently delivered and pregnant women, respectively.

At baseline, ∼15% of recently delivered and pregnant women in both the nutrition-focused and standard MNCH groups reported that someone in the household received cash, food, or other types of social assistance. At endline, 24.3% of recently delivered women in the nutrition-focused MNCH group received such assistance compared to 33.6% in the standard MNCH group (*P* < 0.05). At endline for pregnant women, there was no difference (i.e., 30.3% for the nutrition-focused MNCH group and 31.3% for the standard MNCH group). At both baseline and endline, about one-third of recently delivered and pregnant women in both the nutrition-focused and standard MNCH groups reported that someone in the household received a microcredit loan. A significantly greater percentage of husbands in the nutrition-focused MNCH group, compared to the standard MNCH group, reported being able to buy 11 of the 12 food items queried regularly at endline (**Table 3**).

**TABLE 3:** Percentage of husbands able to buy foods regularly (≥3 times/wk) at endline^1^

Food items	Nutrition-focused MNCH	Standard MNCH
Eggs	95.7***	78.7
Fish	97.3**	84.8
Meat	76.1***	39.9
Dark green leafy vegetables	98.2*	93.0
Yellow/orange vegetables (carrots, pumpkin, sweet potato, etc.)	86.5***	47.5
Yellow/orange fruits (papaya, mango, pineapple, etc.)	70.7***	36.8
Citrus fruits (fruits with vitamin-C, i.e., lemon, guava, *Indian gooseberry*)	61.1*	41.6
IFA tablet	64.0*	42.2
Calcium tablet	62.2*	41.3
Horlicks/other special drinks	15.4*	4.2
Dal/lentils	87.1**	61.2
Other fruits, e.g. apples, grapes, bananas	52.4	38.4

1 *P < 0.05, **P < 0.01, ***P < 0.001. IFA, iron and folic acid; MNCH, Maternal, Neonatal, and Child Health.

The mean social desirability scores were 3.13 ± 1.16 and 2.75 ± 1.25 for the nutrition-focused and standard MNCH groups, respectively (*P* = 0.136 for model adjusted for clustering). The social desirability score was not associated with food insecurity in the nutrition-focused MNCH group (*P* = 0.415), but higher social desirability score was associated with higher prevalence of food security in the standard MNCH group (*P* < 0.001), resulting in a larger difference in prevalence of food security between groups for women with low social desirability score (i.e., 19.7%) than for women with very high score (i.e., 12.6%).

## Discussion

Household food insecurity, as reported by pregnant and recently delivered women, was reduced in areas where the nutrition-focused antenatal care and community mobilization intervention package was implemented. The reduction was consistent across all items, domains, and categories of food insecurity. The nutrition-focused MNCH package, which did not provide food, was delivered during routine antenatal care visits with the use of interpersonal communication and community mobilization that included several components with potential to reduce food insecurity. The package intended to improve knowledge of the decision-makers in the household (e.g., women, husbands, and mothers-in-law) about the importance for fetal development of nutrition for pregnant and lactating women and encouraged husbands in particular to allocate resources to ensure sufficient quantity and quality of food in the households.

We hypothesized that the package would increase the purchase and availability of foods in the household through improved knowledge, altered resource allocation, and use of savings, and that doing so would improve household food security. Our results are consistent with this hypothesis. Compared with the standard MNCH group, in the nutrition-focused MNCH group women reported higher household food security and husbands reported greater likelihood of being able buy food items at endline. Recently delivered women in the standard MNCH group were more likely to report receiving cash, food, or other types of social assistance, which presumably would have improved their food security and thereby would have resulted in underestimation of the impact of the nutrition-focused MNCH package. Our examination of social desirability also suggested that any such bias would have underestimated the impact.

Given that women reported on the status of household food security, an alternative explanation for the impact on food security could be that women specifically benefited from increased purchase and availability of foods rather than all household members, and that their reporting of household food security was influenced by their personal better access to food. Of the 9 questions in the food security scale, 7 asked about “you or any household member,” 1 asked “did you worry that your household,” and 1 asked about “your household;” that is, all questions asked about the household but 8 of 9 included “you” as part of the question. A comparison of men's and women's responses to a 13-item scale measuring food insecurity in Bangladesh found 81% concordance on items and 69% concordance on classification of food insecurity into terciles with no overall bias (26). These results, however, do not provide information about whether men and women would respond similarly if there were differences in household food insecurity as a result of an intervention. In northern Burkina Faso where there was strong seasonal fluctuation in food insecurity, the patterns of food insecurity over time that were reported by male household heads and women were concordant with reality and with each other (27).

In the nutrition-focused MNCH areas, coverage was high (>90%) for interpersonal communication, supplement provision, and weight-gain monitoring. In these areas, compared to the standard MNCH areas, training quality, knowledge of frontline workers, coverage of services, and quality of interpersonal communication were significantly improved (28). In addition to improved quality and frequency of interventions delivered by frontline workers to women, the husbands of the women were simultaneously engaged in mobilizing household resources to improve food access and to shift social norms by encouraging women to consume more and better foods. These findings are evidence that the integration of nutrition interventions into the MNCH program was feasible and well-implemented, and suggest that exposure to the components of the interventions that might have been expected to reduce food insecurity was high.

The results of this study are consistent with 2 prior studies conducted in the United States showing that nutritional assistance during pregnancy or programming through antenatal care can reduce food insecurity. The Special Supplemental Nutrition Program for Women, Infants, and Children is a nationally funded public-health program implemented by states to provide supplemental foods, referrals to health care, and nutrition education. A longitudinal study of the program found that, among women with prenatal severe household food insecurity, earlier participation (i.e., starting in the first or second trimester) was associated with lower odds of any postpartum household food insecurity compared to late participation (i.e., starting in the third trimester) (29). In the second study, which used a quasi-experimental design, women who chose to receive antenatal care in a series of group sessions (i.e., group care) rather than meeting with only an individual provider (i.e., standard individual care) were less likely to report food insecurity in late pregnancy and postpartum (12). Among women who were initially food insecure, group-care participants were more likely to become food secure in these 2 periods compared with individual-care participants. Because group antenatal care provided health education and the opportunity for women to share experiences and knowledge, food security may have improved through increases in confidence and skills to manage household resources.

Food insecurity is a concern in all vulnerable populations because it is a powerful stressor that can result in physical hunger, stress, sadness, shame and stigma, social isolation (1), disruptions in family cohesion (30), and altered parenting (4, 6). Food insecurity is also a marker of scarcity (31) and other stressors such as poor sleep and cognitive overload (32), a strong mediator of poverty (33), associated with less use of healthcare services (34), and a driver of a broad set of detrimental nutritional, psychological, social, and health outcomes (1). Therefore, reducing food insecurity during the pregnancy and postpartum periods when women are particularly vulnerable should be a high priority. Integration into ongoing antenatal care of social and behavioral nutrition interventions provides a potentially effective means to do so, without incurring the high costs of setting up new service-delivery channels and of providing supplemental food, in populations where limited resources can be directed towards accessing adequate and appropriate foods. Future studies are warranted in different contexts of the comparative and cost effectiveness of interventions to reduce food insecurity in the pregnancy and postpartum periods.

## Notes

Supported by the Bill & Melinda Gates Foundation, the Canadian Department of Foreign Affairs, Trade and Development, through Alive & Thrive, managed by FHI 360, and the CGIAR Research Program on Agriculture for Nutrition and Health, led by the International Food Policy Research Institute. Role of Funding Alive & Thrive, represented by co-authors TS, ZM, SA, and BA, participated in the study design but not in the data collection or analysis. Alive &Thrive provided specific inputs to the manuscript regarding intervention design and provided feedback on the interpretation of results. Freedom to publish the study findings was protected contractually in the agreement between the respective funding sources and IFPRI. All final decisions on the manuscript were made by the researchers.

Author disclosures: EAF, PHN, TS, ZM, BA, SA, and PM, no conflicts of interest.
